# Systems view of *Bacillus subtilis* pellicle development

**DOI:** 10.1038/s41522-022-00293-0

**Published:** 2022-04-12

**Authors:** Mojca Krajnc, Polonca Stefanic, Rok Kostanjšek, Ines Mandic-Mulec, Iztok Dogsa, David Stopar

**Affiliations:** 1 Department of Microbiology, Biotechnical Faculty, University of Ljubljana, 1000 Ljubljana, Slovenia; 2 Department of Biology, Biotechnical Faculty, University of Ljubljana, 1000 Ljubljana, Slovenia

**Keywords:** Biofilms, Bacteriology

## Abstract

In this study, we link pellicle development at the water–air interface with the vertical distribution and viability of the individual *B. subtilis* PS-216 cells throughout the water column. Real-time interfacial rheology and time-lapse confocal laser scanning microscopy were combined to correlate mechanical properties with morphological changes (aggregation status, filament formation, pellicle thickness, spore formation) of the growing pellicle. Six key events were identified in *B. subtilis* pellicle formation that are accompanied by a major change in viscoelastic and morphology behaviour of the pellicle. The results imply that pellicle development is a multifaceted response to a changing environment induced by bacterial growth that causes population redistribution within the model system, reduction of the viable habitat to the water–air interface, cell development, and morphogenesis. The outcome is a build-up of mechanical stress supporting structure that eventually, due to nutrient deprivation, reaches the finite thickness. After prolonged incubation, the formed pellicle collapses, which correlates with the spore releasing process. The pellicle loses the ability to support mechanical stress, which marks the end of the pellicle life cycle and entry of the system into the dormant state.

## Introduction

Bacteria may adapt to different habitats and niches in the ecosystem^1^ or even create one that best suits their needs by forming biofilms^2^. Nonhomogeneous structures, such as biofilms, are essential components of the ecosystem dynamics that allow for better survival of bacteria in closed systems^2^. The switch from planktonic to biofilm lifestyle is a major event that requires massive metabolic restructuring and the formation of mechanically coupled cell structures that are not existent in planktonic growth^3^. It has been discovered only recently that individual bacteria dispersed in planktonic suspensions are already weakly mechanically coupled^4^. The coupled bacterial structures allow for mechanical interconnections between the individual cells and coordinated motion of a group of bacteria that are up to 100 μm apart. Although such structures are generally not considered to be biofilms they have several characteristics which are of key importance in biofilm formation. The individual cells are surrounded by a self-made extracellular matrix. The matrix behaves as weak viscoelastic fluid and increases in strength with increasing bacterial density. Though coupled, the individual cells can break from the matrix and swim in the suspension^4^. The transition from the weak viscoelastic structure in the plankton to a strong pellicle structure at the interface is poorly understood.

With an increase in plankton population size, the conditions in the bacterial suspension usually deteriorate and bacteria are forced to find alternative habitats within the system to survive. One major determinant that fine-tunes the distribution of bacteria in the water column is oxygen^5^. Oxygen solubility is dependent on temperature, solute concentration, and when in equilibrium with air, barometric pressure^6^. It was shown that in a typical growth medium supplemented with 50 mM glucose there is not enough oxygen to support a fully aerobic growth and *B. subtilis* become oxygen-limited^7^. Oxygen diffuses to the growth medium from the water–air interface and creates a gradient in the water column^8^. The cells sense the oxygen concentration gradient, swim in the direction up a gradient, and accumulate at the water–air interface. The water–air interface provides a very favourable environment for aerobic microorganisms since they can access high oxygen concentrations as well as nutrients from the medium. The interface colonization is influenced by flagellum-based mobility, surface tension, Brownian motion, Van der Waals attractive forces, gravitational forces, surface electrostatic charge, or hydrophobic interactions^9^. The bacterial accumulation at the interface, however, is equivalent to an increase in fluid density and cells begin to sink. In such a situation, bioconvection begins as a gravitational Rayleigh–Taylor instability^8^. During fully developed bioconvection, oxygen-charged water from the interface convects downward, and *Bacillus subtilis* swim toward a self-generated complex convection-dependent gradient. This is a temporal solution to an increased oxygen demand with a large expenditure in energy for swimming^8^. Bioconvection does not allow the formation of large pellicle structures that are regularly observed on the surface of bacterial suspensions^10^. To stay at the water–air interface, *B. subtilis* must produce mechanical support for a growing bacterial mass at the interface that prevents the sinking of the pellicle^11–15^.

Extracellular polymeric substances (EPS) play the main role in the transition to the biofilm^12,16^ by allowing cells to connect and interact with each other, neutralize or bind antimicrobial agents^17^, provide mechanical stability^18^, and protect cells from predators and rapid extreme changes in the environment^19,20^. EPS allows non-motile long cell chains to adhere to each other^13,14,21^ and is crucial in cell cluster enlargement and formation of complex multicellular communities and different patterns of cell differentiation^22–24^. The entry into the biofilm state involves strict transcriptional regulation of genes responsible for the synthesis of the extracellular matrix components^15,21,25,26^.

In general, biofilms are viscoelastic mechanical structures that allow bacteria to adapt to mechanical stresses^27,28^. Mechanical compression due to constrained biofilm expansion may trigger instabilities that result in out-of-plane deformation and wrinkle formation^29–33^. The viscoelastic behaviour of the biofilm develops due to the production of extracellular material composed of polysaccharides, proteins, lipids, and eDNA^14,34,35^. In *B. subtilis* biofilms, protein TasA and exopolysaccharide EpsA-O have been implicated in biofilm formation^14,21^. TasA is an amyloid-forming protein^34,36^ encoded on the *tapA-sipW-tasA* operon, has a broad spectrum of antibacterial activity^37^, and plays a key role in the spore coat assembly and spore germination process^38^. TasA can form fibrils that are resistant to severe physiochemical conditions^34^ and are important in strengthening and stabilizing the *B. subtilis* biofilm^14,21,34,38,39^. The polysaccharide EpsA-O is synthesized by several enzymes encoded on the 15-gene *epsA-O* operon. Precursors for polysaccharide formation are nucleotide sugars, primarily UDP-glucose and UDP-galactose^22–24,27^. EpsA-O polysaccharide is essential for the formation of a robust biofilm and the integrity of mature biofilm^11–15,21^.

There are only a few direct studies of the temporal dynamics and mechanical stability in growing bacterial pellicles^40–42^. In most studies, the mechanical properties and morphology of the *B. subtilis* pellicles were monitored indirectly, *ex* *situ*, focusing only on the water–air interface without taking into account the events in the bulk of the liquid^13,32,43,44^. Studies show that mechanical stability and rheological characteristics of pellicles depend significantly on bacterial strain/species^44,45^. *B. subtilis* pellicles are viscoelastic materials when they are under compression or in response to small shear deformations^43,46^, and viscoplastic materials if they are treated with larger shear deformations or are under applied tension^43^. Viscoelasticity of the biofilm is determined by bacterial cells density, the extracellular matrix^28,47^, cell chain entanglement, and crosslinking^48^. The study of the rheological properties of the interfacial pellicles has greatly improved our understanding of pellicle structures^41,42^. With interfacial rheology, Rühs et al. monitored the mechanics of the newly formed pellicle of *B. subtilis* and a mutant strain that did not produce surfactin in the LB medium. It was found that the decrease in elasticity, as well as the decrease in surface tension, was most likely due to surfactin^41^. Recently, Pandit et al. used interfacial rheology to monitor mechanical and macroscopic changes in the first 24 h of *B. subtilis* pellicle formation after vitamin C supplementation^42^. The addition of vitamin C caused stagnation in pellicle formation and lower storage modulus.

In this study, we are focusing on developmental stages that accompany the lifecycle of *B. subtilis* PS-216 pellicle in a batch growth system. The wild type, naturally competent strain, PS-216 was isolated from the riverbank soil^49^, it has a known genome sequence^50^, forms robust pellicles at the water–air interface^25,51,52^, and biofilms on plant roots^53^. The strain has been adopted as a model system to investigate biofilms, bacterial sociobiology including quorum sensing^25,51,52,54–56^, sporulation^57^, and genetic competence for transformation^50,58–60^. By combining the real-time interfacial rheology and confocal laser scanning microscopy (CLSM), we correlated morphological changes (aggregation status, filament formation, pellicle thickness, spore formation) at the water–air interface with mechanical properties of the growing pellicle, including storage (*G*″) and loss (*G*′) moduli, and their ratio (tan *δ*). Since pellicle structure is an integral part of the batch system we have linked the events at the water–air interface with the vertical distribution and viability of the individual bacterial cells in the water column and at the solid-water interface at the bottom of the batch system, which is a unique perspective on pellicle development. To determine the effect of specific EPS on pellicle formation we have applied three mutant strains: the strain with the inactivated *epsA-O* operon, the strain with impaired production of the TasA protein, and the double mutant unable to produce both EpsA-O and TasA.

## Results

### Evolution of mechanical properties during pellicle formation

**Fig. 1:**
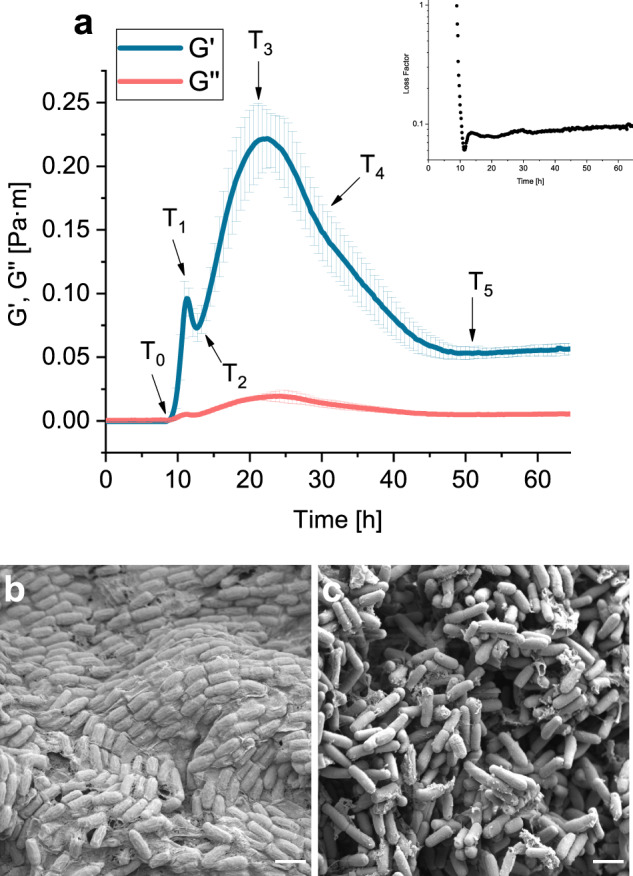
Pellicle mechanical properties. **a** Interfacial storage modulus (*G*′), loss modulus (*G*″), and loss factor (inset) during pellicle formation at the water–air interface in MSgg medium for *B. subtilis* PS-216 wild type strain. Results are presented as mean ± standard deviation from six independent biological measurements. *T*_0_–*T*_5_ denotes key events in viscoelastic behaviour during pellicle formation. *T*_0_ represents the initial increase of storage modulus; *T*_1_ is the local maximum, *T*_2_ local minimum, *T*_3_ global maximum, *T*_4_ inflection point, and *T*_5_ represents the values of storage and loss moduli after pellicle collapse. Viscoelastic curves were obtained by interfacial rheology with a biconical measuring system. **b** Scanning electron micrographs of the top layer of the wild type pellicle in contact with the air, and **c** bottom layer of the wild type pellicle in contact with the liquid MSgg medium. Scale bars represent 2 µm.

To obtain information on the mechanical properties of newly formed pellicles of *B. subtilis* PS-216 we used interfacial rheology to monitor viscoelastic properties as they develop in situ during the incubation. The viscoelastic properties of emerging pellicles changed significantly and several key events can be observed in viscoelastic curves (Fig. 1a). Pellicle formation started at around 8 h of incubation, as indicated by a small increase of interfacial storage and loss moduli (*T*_0_). After 9 h of incubation, there was a sudden increase in the pellicle storage modulus and a modest increase in loss modulus. Around 11 h of incubation, the increase in the storage modulus was shortly interrupted (*T*_1_) and the strength of the pellicle structure decreased significantly reaching the local minimum after 12.5 h of incubation (*T*_2_). This was followed by a significant increase in storage modulus up to 23.5 h of incubation when the global maximum of the storage modulus was observed (*T*_3_). After this point, the storage (*G*′) and loss (*G*″) modulus decreased (*T*_4_), initially faster and then slower to 50 h of incubation when storage and loss modulus remained stable (*T*_5_). As indicated by the loss factor (tan *δ* = *G*″/*G*′) the pellicle behaved as viscoelastic fluid during the formation phase (Fig. 1a—inset). At *T*_0_, pellicle material reached sol/gel transition point and remained thereafter in a gel state where the storage modulus was consistently higher than the loss modulus. The loss factor of a strong pellicle of approximately ~0.1 is consistent with low stringiness and relatively firm gel material with brittle character. The maximum interfacial *G*′ value for *B. subtilis* PS-216 in MSgg medium (0.25 Pa·m) is comparable to the interfacial *G*′ values of 0.20 Pa·m for *B. subtilis* PY79^41^ and also to the interfacial *G*′ values of 0.30 Pa·m for *B*. *subtilis* 3610 in LB^42^. The values of interfacial *G*″ modulus were an order of magnitude lower than *G*′ and similar to the ones reported in the literature^41,42^.

The cell organization of the mature pellicle at *T*_3_ was significantly different at the two interfaces as indicated by SEM (Fig. 1b, c). The top face of the pellicle, which was exposed to air, had orderly arranged bundles of cells that were embedded in the extracellular matrix (Fig. 1b). The bottom side of the pellicle, exposed to MSgg medium, had disorganized and scattered cells in different orientations (Fig. 1c). The cells were covered with patches of extracellular material that were different from the matrix in the top face.

Macroscopic morphology of the resulting pellicles in the IRS (interfacial rheological system) vessel after 24 and 64.5 h of incubation is given in Supplementary Fig 1. After 24 h, the pellicle was white and changed to yellow-brown at the end of incubation (65 h). When the measuring system was lifted after 24 h the pellicle remained compact, whereas after 65 h it disintegrated. The pellicle wrinkling increased with incubation. The macroscopic pellicle morphology after 65 h was similar to the morphology of *B. subtilis* DV1 strain after 67 h of incubation observed by Trejo et al.^32^. Pellicle showed the presence of large vertical structures of localized folds coexisting with wrinkles.

### Matrix components influence the evolution of pellicle mechanical properties

**Fig. 2:**
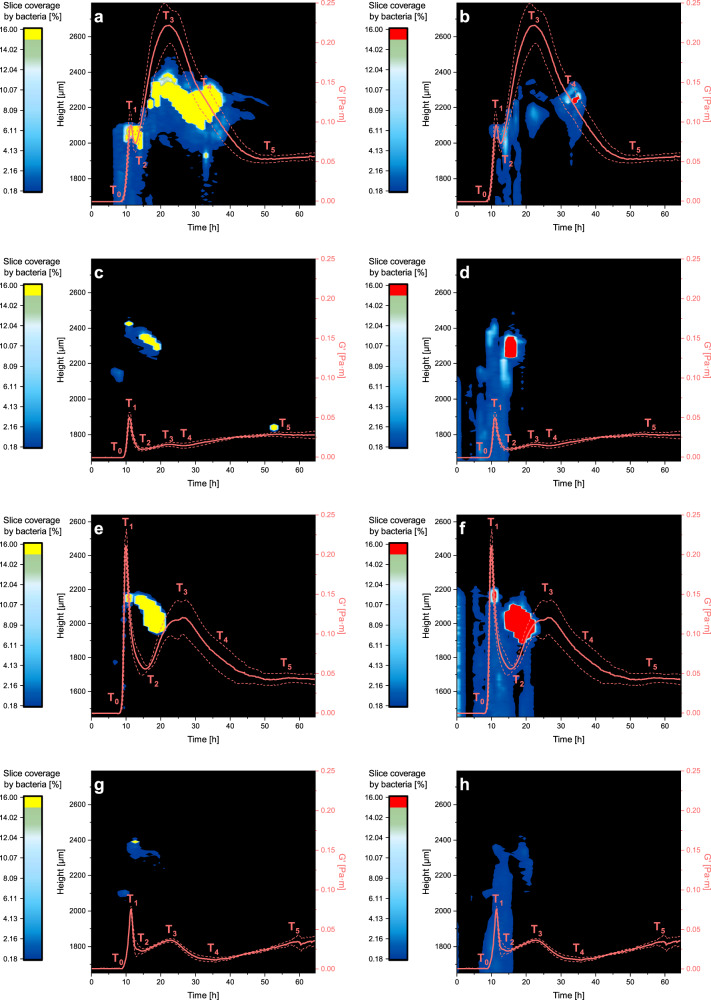
The correlation between storage modulus and vertical bacterial density at the water–air interface. The images are presented as a combination of data obtained by CLSM and interfacial rheology method showing both viable (**a**, **c**, **e**, **g**) and dead cells (**b**, **d**, **f**, **h**) for *B. subtilis* PS-216 wild type strain (**a**, **b**), Δ*tasA* (**c**, **d**), Δ*epsA-O* (**e**, **f**) and Δ*epsA-O* Δ*tasA* (**g**, **h**) mutant. Values above 16% are in the same colour. *T*_0_–*T*_5_ denotes key event points where a drastic change in viscoelastic behaviour occurred. In *tasA* mutant and in the double mutant, the inflection point *T*_4_ turned into the second minima. Viscoelastic curves are presented as mean ± standard deviation from 3 to 6 independent biological measurements. One of 3–6 qualitatively similar replicates of vertical bacterial density is shown.

To test whether extracellular matrix polymers change pellicle dynamics we have grown pellicles of mutants, which do not produce the extracellular protein TasA and polysaccharide material encoded in *epsA-O* operon. The Δ*tasA* mutant (Fig. 2c, d) formed pellicles but the storage and loss moduli were significantly lower compared to the wild type pellicle (Fig. 2a, b). The formation of the pellicle started slightly later as in the wild type (Supplementary Table 2). The initial rise of the storage modulus at *T*_1_ was 50% lower than in the wild type, there was no peak at *T*_3_, and elasticity at *T*_5_ was much lower than in the wild type. The viscous modulus (loss modulus (*G*″)) was lower than in the wild type. Overall, this suggests that TasA protein contributes significantly to the elastic properties of the wild type pellicle. The bacterial strain defective in *epsA-O* extracellular polysaccharides (Fig. 2e, f) had a different effect on the pellicle mechanical properties. The formation of the pellicle started earlier than in the wild type, the storage modulus was larger at *T*_1_, but much lower at *T*_3_ compared to the wild type. The loss modulus (*G*″) curves were comparable in the EpsA-O mutant strain and the wild type. The storage modulus for a double mutant (Δ*epsA-O* Δ*tasA*) was different from either of the single mutants (Fig. 2g, h). As the pellicle thickness of the mutants was significantly different during the pellicle growth (Fig. 5) the storage modulus was normalized to a maximum pellicle thickness. In normalized storage moduli (Supplementary Fig 2) the strength of the double mutant pellicle stands out. Although absolutely this pellicle was the weakest, the normalized elasticity was rather large.

### Temporal correlation between viscoelastic behaviour and development of pellicle morphology

There was no accumulation of viable cells at the interface up to *T*_0_ (Fig. 2a). The accumulation of viable cells at the interface increased between *T*_0_ and *T*_1_, when a band of interfacial pellicle formed, correlated with the increase in pellicle interfacial elasticity. The pellicle reached the thickness of approximately 100 μm when after *T*_2_ a sudden relocation in pellicle interface position was observed. The pellicle moved vertically for approximately 200 μm, which correlated with a major increase in pellicle elasticity. Up to *T*_3_, the volume of the pellicle expanded and pellicle thickness reached ~300 μm. This was followed by a sudden collapse of the pellicle when the number of viable bacteria at the interface dramatically decreased. Although the pellicle was still predominantly composed of viable bacteria there was a significant fraction of dead bacteria present (Fig. 2b). It is important to note that at *T*_2_ when the elasticity of the pellicle reached the local minimum the number of dead bacteria increased significantly. Similarly, an increase in the fraction of dead bacteria correlated with the decay of the pellicle at *T*_3_ and *T*_4_.

The mechanical properties of the mutant pellicles also correlated with bacterial density, viability, and pellicle morphology. The bacterial densities and pellicle thickness were significantly lower in the Δ*tasA* mutant compared to the wild type (Figs. 2c and 5b). Pellicle produced by the Δ*tasA* mutant decayed earlier and the increase in the fraction of dead cells (Fig. 2d) correlated with the pellicle elastic breakdown. The Δ*epsA-O* mutant, on the other hand, had thicker pellicles than the Δ*tasA* mutant (up to 220 μm) (Fig. 5c). The elasticity breakdown at *T*_2_ (Fig. 2e) correlated with the massive increase in the fraction of dead cells (Fig. 2f) and prevented a full elastic development between *T*_2_ and *T*_3_. The fractions of both viable and dead cells were significantly reduced in the double mutant, the thickness of the pellicle was very low (Fig. 5d), and the pellicle at the interface existed only for a short period of time (Fig. 2g, h). Images of mature pellicles that developed in the microtiter plates are given in Supplementary Fig 3. The wild type and the Δ*tasA* mutant pellicles appear similar. In both Δ*epsA-O* and the double mutant, bacteria produce a patched pellicle structure. The SEM micrographs of the three mutant pellicles are given in Supplementary Fig 4. The top surfaces of the three mutants were remarkably different. The Δ*tasA* mutant was covered with a continuous thin matrix material. Cells were organized nose to tail in bundles similar to the wild type. The Δ*epsA-O* mutant pellicle surface exposed to air had disorganized cells that were covered with a flake-forming matrix material that was attached to the cell surface with rope structures. In the double mutant, cell density was much lower and several cells had filament morphology. They were embedded in a fractal-like porous extracellular material. The pellicle’s bottom faces of the Δ*tasA* and Δ*epsA-O* mutants were similar. Cells were disorganized and connected via a rope extracellular material. In addition, in the Δ*tasA* mutant, there was more lace-resembling extracellular material.

### The systems approach to monitor pellicle development

**Fig. 3:**
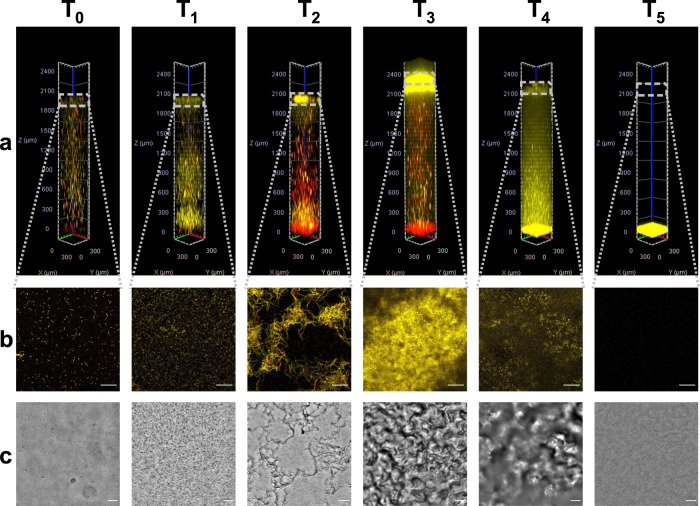
Systems temporal dynamics. **a** The vertical distribution of *B. subtilis* PS-216 wild type bacterial cells in the entire water column at identified key viscoelastic events (*T*_0_–*T*_5_). Viable yellow cells are constitutively expressing YFP, while the red cells with compromised membranes were stained with propidium iodide. The columns were constructed by stacking optical slices obtained by CLSM using the Zeiss Zen Blue program. A total of 45 slices taken 60 μm apart were used to construct the image. A maximum intensity projection is shown. **b** A single optical slice of the pellicle at the water–air interface. Scale bar represents 50 μm. **c** Microscopic images obtained by differential interference contrast light microscopy (DIC) microscopy at the water–air interface. Scale bar represents 50 μm.

To better understand the pellicle development, we simultaneously monitored bacteria in the pellicle, water column, and at the bottom of the microtiter plate well (Fig. 3). During the pre-pellicle formation stage (*T*_0_) cells were homogeneously dispersed in the water column. The number of cells per unit volume at the water–air interface was not larger than in the water column. No additional structure at the interface could be observed with DIC microscopy. After *T*_0_, a redistribution of cells within the water column was detected (Fig. 3a). The viable cells either moved towards the surface of the liquid medium or to the bottom of the water column forming respective biofilm structures. At the water–air interface, viable cells began to form micro aggregates as observed on fluorescence and DIC micrographs (Fig. 3b, c). This correlated with an increase in pellicle elasticity. At *T*_2_, the entangled bacterial filaments formed and there was a clear partitioning of the viable cells to the interface pellicle and of the dead cells to the bottom of the water column. It is interesting to note that a significant fraction of cells in the water column at *T*_2_ was dead, according to PI staining. At *T*_3_, when the pellicle achieved maximum elasticity a robust and confluent pellicle was visible at the interface and composed predominantly of viable cells. Pellicle wrinkles were observed on DIC micrographs. There was dense sediment of dead cells at the bottom of the water column. A major restructuring occurred at *T*_4_ when the majority of live cells were in spore developmental or spores dispersed process, and most of the water column was yellow with YFP fluorescence emitting spores and YFP debris that increased background colouring (Supplementary Fig 5). The sediment turned yellow due to the deposition of the labelled material. At *T*_5_, the water column has cleared of cells and fluorescently labelled material and spores which sedimented completely to the bottom of the water column. The microscopic examination of the suspension at *T*_4_ and *T*_5_ at higher magnifications indicated that spores represented the majority of particles in the system. The fraction of spores was 95% or higher.

**Fig. 4:**
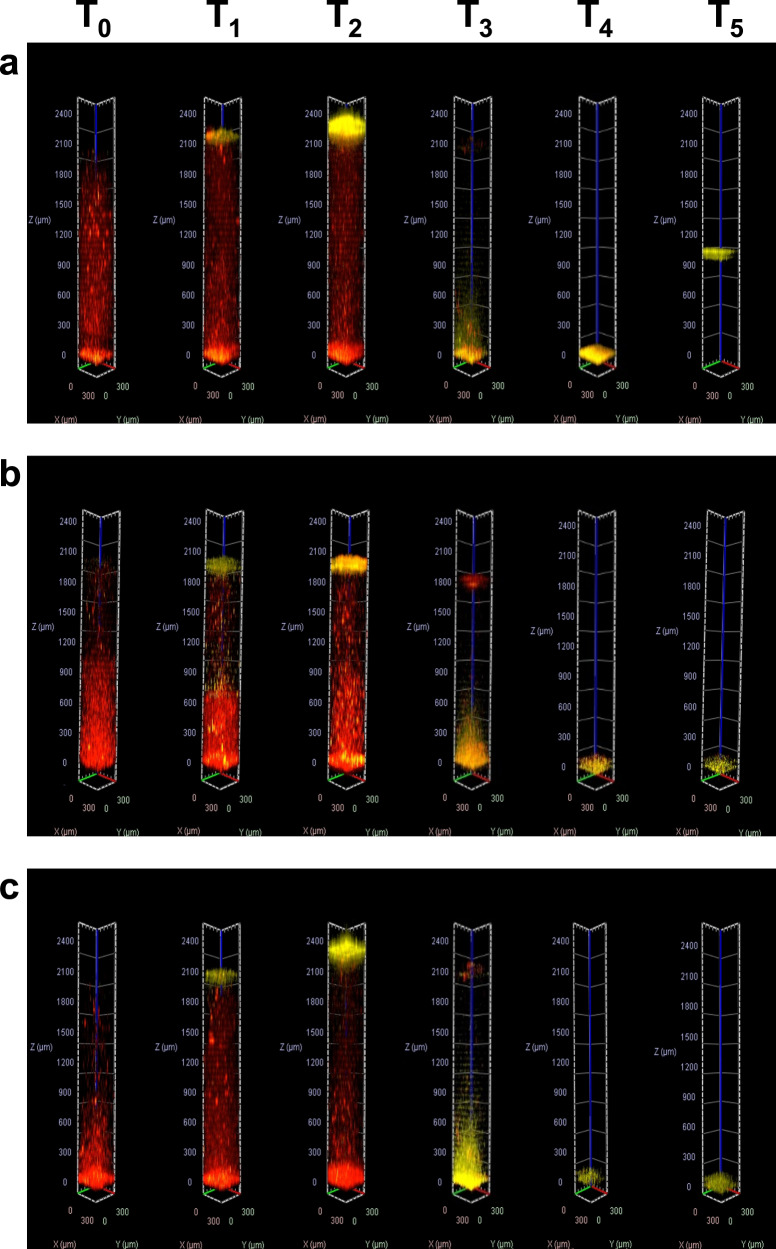
*Systems temporal dynamics of mutant strains*. The vertical distributions of *B. subtilis* PS-216 Δ*tasA* (**a**), Δ*epsA-O* (**b**), and Δ*epsA-O* Δ*tasA* (**c**) mutant cells at identified key viscoelastic events (*T*_0_–*T*_5_). The columns were constructed by stacking optical slices as described for the wild type in Fig. 3.

The system developed quite differently in mutant strains of *B. subtilis* PS-216 (Fig. 4). The most obvious difference was a much larger fraction of the cells with compromised membranes in the water column in the pre-pellicle formation stage. The temporal distribution of viable and membrane compromised cells along the vertical profile during system development is shown in Supplementary Fig 6. This suggests that mutants experienced higher physiological stress compared to the wild type. As expected, in all mutants less pellicle formed at the interface, the pellicle was thinner and weaker and did not climb the walls of the wells as significantly as in the wild type strain. We have observed that all mutant sediments were composed of predominantly dead cells, and formed much earlier than in the wild type. Surprisingly, in the Δ*tasA* mutant at T_5_, sediment at the bottom of the column detached and had a neutral buoyancy in the middle of the water column (Fig. 4a). In the case of the Δ*epsA-O* mutant (Fig. 4b), the system maturated later compared to the wild type and formed a more robust pellicle than the double mutant strain (Fig. 4c).

**Fig. 5:**
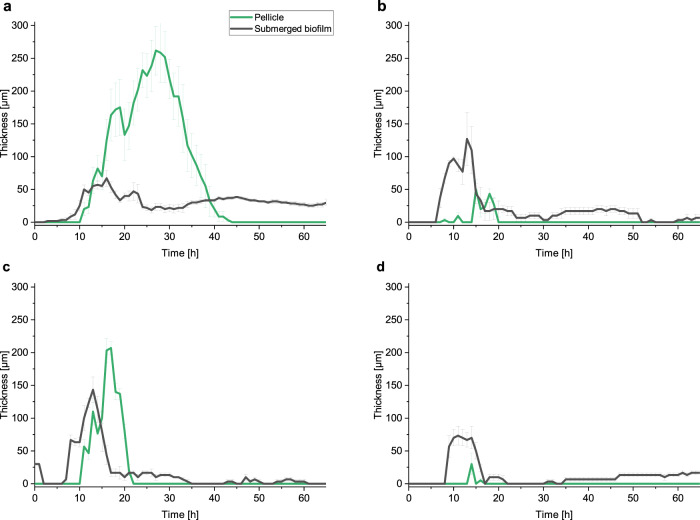
*Pellicles and submerged biofilms thicknesses*. Temporal variations of pellicle and submerged biofilm thicknesses for *B. subtilis* PS-216 wild type (**a**), Δ*tasA* (**b**), Δ*epsA-O* (**c**), and Δ*epsA-O* Δ*tasA* (**d**) mutant. Results are presented as mean ± standard error from 3 to 6 independent biological measurements.

In addition, we have determined the temporal evolution of biofilm thicknesses at the water–air interface and at the bottom of the well (Fig. 5). The thickness of the pellicle correlated with mechanical properties in all tested strains. The maximum thickness was 300 μm for the wild type, 220 μm for Δ*epsA-O* mutant, 50 μm for Δ*tasA* mutant, and 40 μm for the double mutant. The submerged biofilm formed in all strains was consistent with the observation by Bridier et al. ^61^. The submerged biofilm appeared earlier than the pellicle at the water–air interface (Fig. 5). This was more pronounced for the mutants. For Δ*tasA* and the double mutant, the thickness of the submerged biofilm was larger than the thickness of the pellicle. After the disintegration of the pellicle, the material sedimented and contributed to the thickness of the submerged biofilm.

## Discussion

Although extensive literature on *B. subtilis* biofilms is available^14,21,24,28,39,42,62–64^, a systematic study addressing temporal dynamics of *B. subtilis* life cycle involving pellicle formation and disintegration in the water column is lacking. Here we present a systematic in situ study of pellicle lifecycle in a batch system composed of a single species *B. subtilis* PS-216. We have compared morphological and mechanical changes in the pellicle in real-time with changes in the water column and sediment to provide a systems view of key events in the pellicle lifecycle. Six key events have been identified (*T*_0_–*T*_5_) in *B. subtilis* pellicle formation that are accompanied by a major change in viscoelastic and morphologic behaviour of the pellicle.

The most important result of this study is that the formation of a pellicle at the water–air interface cannot be studied in isolation from the rest of the system in which pellicle-forming bacteria grow. In our experiments, the system permitted only air exchange and bacteria could select between three major habitats: water suspension, solid–water, and water–air interface. It appears that cells before *T*_0_ were preferentially located in the suspension and did not have a favoured stratum. To form a pellicle *B. subtilis* needs to build a supporting floating structure where cells are glued together with extracellular polysaccharides, proteins, lipids, and nucleic acids^14,35,36,65^. It was previously demonstrated that *B. subtilis* biofilm can make glue components already in the bacterial suspension before the formation of the pellicle at the interface^4^. It was shown that after 8 h of incubation the extracellular material can fill most of the extracellular space in suspension, which coincides with the first viscoelastic structures measured at the interface.

During the formation of an initially weak pellicle at the water–air interface, a major redistribution of bacteria in the water column took place, as though bacteria had to choose to move either to the water–air interface or to the solid-water interface at the bottom of the water column. While the first strategy proved to be successful, the latter was equivalent to sedimentation to the bottom of the well with the majority of cells there being dead. In the double mutant that did not produce TasA and EpsA-O extracellular polymers, the timing of the first viscoelastic pellicle structure at the interface was not different from the wild type. This is consistent with earlier findings where the production of TasA and EpsA-O was low at low cell densities and increased significantly only during the transition to the stationary growth phase^3,4^, and suggest that these extracellular polymers are less important in the initial stages of the pellicle formation process.

Newly accumulated cells at the water–air interface continued to grow but cell division was impaired, which caused the formation of long bacterial filaments at the water–air interface (Fig. 3, *T*_2_). The entanglement of filaments marks a transition to a stronger pellicle structure and provides a scaffold to which newly synthesized extracellular matrix components were added^62^. During the filament formation stage, we noticed that a large number of cells died. This correlated with pellicle restructuring and may together with surfactin release^31^ lead to a temporal decrease in pellicle elasticity.

To form strong mature biofilms, *B. subtilis* must remodel its metabolism by producing TasA and EpsA-O extracellular polymers^14,21,34,38,39^. It has been shown, by transcriptome, proteome, and metabolome analyses that matrix component synthesis is upregulated during biofilm formation^3^. The upregulation of extracellular matrix genes *epsA-O*, *tapA-sipW-tasA*, and *bslA* operons and the increase in the levels of extracellular matrix biosynthetic intermediates UDP-Glc and UDP-GlcNAc were concurrent with the upregulation of the TCA cycle. TasA production was induced after 12 h of incubation, which corresponds to the pellicle stage *T*_2_ and transition to a stronger pellicle. Interestingly, the *epsA-O* and *tapA-sipW-tasA* operons displayed only a transient increase in expression (peaking at 16 h) rather than the sustained increase that might be expected for extracellular matrix genes in growing biofilms^3^. Despite the transient transcriptional upregulation of *tapA-sipW-tasA*, a sustained increase in protein levels of TasA that persisted throughout biofilm development was observed^3^. It has been demonstrated by Magic-angle spinning NMR applied in vivo to *B. subtilis* biofilms that newly synthesized soluble TasA protein restructures biofilm to protease-resistant biofilm^36^. These results are in excellent agreement with ours and suggest that TasA protein is the most important extracellular matrix mechanical component that starts the transition from weak to strong pellicle (i.e. from *T*_2_ to *T*_3_). TasA is necessary for the formation of elastic pellicles and wrinkle formation in fully mature pellicles^14^. Although in the absence of TasA pellicle could still form, it was weak, thin, and decayed fast (Fig. 5b). In addition to TasA protein, extracellular polysaccharide EpsA-O was also important in a transition to the strong pellicle. In the absence of EpsA-O, the elasticity of the pellicle decreased by 55% compared to the wild type. When both extracellular polymers (TasA and EpsA-O) were missing in the double mutant the pellicle was extremely weak and fragile, which agrees with the literature^13^. The pellicle of the double mutant nevertheless formed at approximately the same time as in the wild type, but it remained brittle and did not mechanically evolve, due to the lack of fortification with TasA and EpsA-O polymers.

With the formation of the strong pellicle, the system changed profoundly. The most viable place for bacteria in the system becomes the pellicle. The pellicle at the interface likely acted as a barrier for oxygen penetration to the interior of the water column. However, the more pellicle grew in thickness fewer nutrients could be obtained from the growth medium and it became more likely that spore formation will occur^25,63^. The spores in pellicles reaching maturity have been observed by Spacapan et al.^25^. At approximately the same time, we have observed an increased fraction of the dead cells. It is known that in the last stage of spore formation the mother cell undergoes lysis^64^, which releases enzymes in the environment that could decrease pellicle strength. How this weakens the pellicle structure is currently unknown. From a spore perspective, however, weakening of the matrix is useful as it helps to release spores and enables their dispersion. The YFP material either in spores or in cell fragments begins to sediment to the bottom of the water column and finally, at *T*_5_, when the system breaks down the fluorescence material is cleared from the water column and all the YFP material, including spores, was present in the compact sediment. The system likely went into a dormant state.

The breakdown of the pellicle at *T*_5_ was not observed in all model systems. For instance, in the rheological IRS measuring system, the pellicle remained attached to the measuring system (Supplementary Fig 1), whereas in microtiter plates it sank to the bottom (Fig. 3). The pellicle in the IRS measuring system was growing in the narrow interfacial gap between the biconical measuring system and the wall of the metal vessel (5.3 mm). On the other hand, the pellicles in the 12-well microtiter plates were grown in a well with a diameter *d* = 23 mm. The two systems followed the same path up to *T*_4_, but the outcome of the pellicle decay was different. In the much smaller gap in the IRS system, the pellicle remained attached to the walls and the surface of the measuring device, whereas in four times larger microtiter plates it broke down after *T*_4_ when the storage modulus of the decayed pellicle significantly decreased. The weakened pellicle was no longer able to support the bacterial mass. Bending of the surface caused increased shear force and bending moments in the plane of the pellicle, which exceeded the pellicle’s ability to support stress. The pellicle in the middle of the well overturned and sediment to the bottom of the water column.

In conclusion, the pellicle formation of *B. subtilis* at the water–air interface in a batch growth system is a multifaceted response to a changing environment induced by bacterial growth that causes population redistribution within the system, reduction of the viable habitat to the water–air interface, cell development, morphogenesis, and build-up of mechanical stress supporting structure. Eventually, the increasing biomass of the pellicle at the interface reduces nutrient availability and results in a finite pellicle thickness. The pellicle destruction correlates with the spore release and the pellicle’s ability to support mechanical stress, which marks the pellicle disintegration and the entry of the system into dormancy.

## Methods

### Bacterial strains, growth medium, and growth conditions

To monitor mechanical properties and morphology *B. subtilis* PS-216 wt^49^ and its derivative strains were used in this study (Supplementary Table 1). Parent strains were tagged with a *yfp* gene linked to a constitutive promoter p43 using pEM1071 plasmid^59^, and chromosomal DNA carrying *yfp* reporter gene linked to p_hyper_cl_0_3 ZK4101^66^, inserted at the *sacA* and *amyE* locus, respectively. Strains knockouts in ∆t*asA* (Sp) and ∆*epsA-O* (Tc) were obtained by transforming the parent strain with chromosomal DNA obtained from strain *B. subtilis* 3610 DL963^14^ and *B. subtilis* 3610 ZK4300^67^, respectively (Supplementary Table 1). The chromosomal DNA (or plasmid) was introduced to the *B. subtilis* using a standard transformation protocol. Bacterial strains were stored at −80 °C. The strains were transferred to LB solid agar plates (tryptone 10 g/l; yeast extract 5 g/l; NaCl 5 g/l; agar 1.5 g/l (w/v)) prior to experiments and incubated for 20 h at 37 °C. Overnight cultures were grown in liquid LB medium (tryptone 10 g/l; yeast extract 5 g/l; NaCl 5 g/l) containing spectinomycin (100 μg/ml), tetracycline (10 μg/ml), chloramphenicol (5 μg/ml) or combination thereof at 37 °C with shaking (200 rpm) for 16 h. To reduce the experimental error, we prepared a stock of bacterial inoculum for each strain grown to late exponential phase (OD_650_ = 0.8 a.u.), aliquoted them in sterile 2 ml micro-centrifuge tubes, and stored them at −80 °C. Cell cultivation was performed in a standard liquid minimal medium MSgg: 100 mM MOPS (3-(*N*-morpholino)propane sulfonic acid); 5 mM K_3_PO_4_; 50 mg/l tryptophan; 50 mg/l phenylalanine; 2 mM MgCl_2_ ∙ 6H_2_O; 0.5% (w/v) sodium glutamate; 0.5% (w/v) glycerol; 700 μM CaCl_2_ ∙ 2H_2_O; 50 μM FeCl_3_ ∙ 6H_2_O; 50 μM MnCl_2_; 1 μM ZnCl_2_; 2 μM thiamine hydrochloride. The pH was adjusted to 7.0. All components of the medium, except thiamine hydrochloride, were autoclaved together at 110 °C. Thiamine hydrochloride was filtered into a sterile bottle and aseptically added after autoclaving.

### Interfacial rheology

To measure the viscoelastic properties of the emerging pellicles, we used a modular oscillating rheometer equipped with an interfacial rheological measuring system (Anton Paar Physica MCR 302 - IRS). The interfacial rheological system included the IRS dish and the associated biconical measuring system with a slope (*α* = 4.988°) and a large circumference to ensure enough contact between the measuring system and the pellicle (diameter 68.162 mm). Pellicles were grown at 37 °C in situ in a special culture dish with a diameter of 80 mm. In all, 34.5 ml MSgg liquid medium in IRS dish was inoculated with 5% thawed inoculum. The Anton Paar RheoCompass software guided the biconical measuring system to detect the position of the interface. Data were collected periodically (every 9 min), at a strain amplitude, *γ*_0_ = 0.1%, angular frequency *ω* = 1 Hz, and at a normal force, *F*_N_ = 0 N. Automatic determination of the normal force allowed the measuring system to adapt to changes in vertical position of the pellicle during the experiment so that throughout the experiment the measuring system was in contact with the surface of a pellicle. In total, 430 measuring points were captured during the 64.5 h of incubation.

The interfacial rheological system used is extremely sensitive and can detect very weak interface structures (the limit of detection of storage (elastic) and loss (viscous) modulus is approximately 0.35 mPa). The raw rheological data were analysed by Anton Paar RheoCompass (ver. 1.25.373) software with the Interfacial Flow Field Analysis method to obtain data on interfacial viscoelastic properties. The theoretical background for the analysis of the measured quantities was described by Soo-Gun and Slattery^68^, Erni et al.^69^, and Tajueloa et al.^70^. Here we present a brief review of the main equations. The linear model for the relationship between interfacial shear stress and interfacial shear rate was first proposed by Boussinesq^71^. Briefly, the Boussinesq number is a dimensionless number to evaluate the influence of the interface drag on bulk drag and is defined as:

1 $${\rm{Bo}} = \frac{{{\rm{Interface}}\;{\rm{drag}}}}{{{\rm{Subphase}}\;{\rm{drag}}}} = \frac{{\eta ^ \ast }}{{\left( {\eta ^{\left( 1 \right)} + \eta ^{\left( 2 \right)}} \right)R_1}}$$

where *η* denotes complex interfacial shear viscosity, *η*^(1)^ denotes lower phase shear viscosity (liquid phase), *η*^(2)^ represents upper phase shear viscosity (gas phase) and *R*_1_ is the radius of the IRS growth dish. Bo >1 means that the influence of the interface over bulk fluid is dominant and that the interface can be considered as a 2D fluid. In this case, complex interfacial shear viscosity can be calculated with the following equation:

2 $$\eta ^ \ast = \frac{{M - \frac{8}{3}R_2^3\left( {\eta ^{(1)} + \eta ^{(2)}} \right)\omega }}{{4\pi R_2^2\omega }}$$

where *M* denotes torque exerted on the disk, *ω* is the angular frequency and *R*_2_ is biconical disk radius, with $\frac{{R_2}}{{H_1}} \to 0$ and $\frac{{R_2}}{{R_1}} \to 0$. This equation is appropriate for highly viscous interfacial films at low shear rates. In oscillation mode, the biconical disk oscillates with constant angular frequency *ω* and strain amplitude *γ*. With a defined deformation (*γ*_s_(*t*) = *γ*_0_·cos(*ωt*)) and with a specific phase shift (*δ*) we can measure the stress response (*τ*(*t*) = *τ*_0_·sin(*ωt* + *δ*)). From this notation, we can define the dynamic complex interfacial shear modulus as:

3 $$G^ \ast \left( \omega \right) = \frac{{\tau _0}}{{\gamma _0}}e^{i\delta } = \left| {G^ \ast } \right|\left( {\cos \delta + i\sin \delta } \right) = G{\prime}\left( \omega \right) + iG{\prime\prime}\left( \omega \right)$$

where *G*′ is the interfacial storage modulus and *G*″ is the interfacial loss modulus. The two moduli can be related to the real and imaginary parts of the complex interfacial shear viscosity (η^*^ = *η*′ − *iη*″) by *G*′(*ω*) = −*ω*·*η*″(*ω*), *G*″(*ω*) = *ω*·*η*′(*ω*), where η^*^ denotes complex interfacial shear viscosity, *η*″ is the elastic and *η*′ the viscous portion. Interfacial storage and loss modulus were used for the characterization of the rheological behaviour of the interface in this study.

### Morphological dynamics of pellicle formation

The morphological dynamics of pellicle formation was monitored by time-lapse CLSM. CLSM is a very powerful and sensitive method to examine the 3D structure of biofilms^72–75^, which allowed us to correlate changes in viscoelastic behaviour with morphological changes in the pellicle. Samples were grown under a microscope in 12-well microtiter plates (Greiner CELLSTAR) with glass lids, which allowed parallel measurement of several samples and thus better statistics of the results obtained. The bottom of the microtiter plates was covered with self-adhesive aluminium foil, ensuring uniform heat transfer throughout the plate. In the middle of each well, a hole was cut into the aluminium foil which enabled access of a laser beam to the sample. To a microtiter well, 1250 µl of MSgg medium and 5% inoculum were added. To determine the proportion of dead bacterial cells in the newly formed pellicles, we added propidium iodide (PI) in a final concentration of 3 μM into every well of the microtiter plate. Propidium iodide binds to the bacterial DNA in cells that have a compromised cytoplasmic membrane. To reduce evaporation, the microtiter plate was coated with silicone paste at contact with the lid and taped with Micropore tape. The microtiter plate was placed into a Heating Insert (Pecon) that maintained a constant culture temperature. The cover of the carrier was set to 38.0 °C, and the lower part of the carrier for microtiter plates at 37.2 °C. In addition to the temperature control system, the microtiter plate carrier was covered with Kapton polyimide adhesive tape, to which we connected the BASETech BT-153 power supply. The electrical voltage was adjusted to 6.3 V. The entire room in which the microscope stands was kept at 25 ± 1 °C during the whole experiment, and relative humidity in the room was kept at around 40–45%. With all these additional settings, we significantly reduced the temperature gradient and consequently improved the profiling quality of the vertical stacks in the water column.

### Microscopy

CLSM was performed with an LSM 800 equipped Axio Observer Z1 (ZEISS) inverted microscope, using a 20 x magnification lens (NA 0.4). The YFP protein signal was excited with a diode laser at a wavelength of 488 nm (green laser), and the PI signal with a diode laser at a wavelength of 561 nm (red laser), and pinhole 97 μm. The used setting of the pinhole improved the acquired emitted light intensity significantly at an optical slice thickness of 11 μm. The intensity of the lasers was changed linearly according to the position in the specimen, i.e. according to the distance from the laser light source to compensate for the light loss (i.e. absorption, scattering). The scanning by green laser started at the bottom of the specimen with an intensity of 1.5% and ended at the top of the specimen with an intensity of 3.5%, while the red laser started with an intensity of 1.0% and ended at 2.6%. At each time interval, the emission light (590–700 nm for PI and 400–590 nm for YFP protein) was recorded on two GaAsP PMT detectors, operating at 800 V. Three image stacks (512 × 512 pixels) were measured for each well. Since the most interesting system dynamics was at the water–air interface and at the bottom of the microtiter wells, we have zoomed into these regions. First, we coarse sliced throughout the specimen (50 slices in steps of 60 μm), then we sampled the bottom of the well (50 slices in steps of 10 μm), and finally the water–air interface (100–150 slices in steps of 10 μm). With the described settings, we have optimized the method so that each sample in the 12-well microtiter plate was scanned once every hour. The duration of the experiment was 65 h. The detailed description of the used settings is in Supplementary Note 1. Using computer tools and algorithms (Fiji ImageJ 1.52p, Zeiss ZEN 3.3 (Blue edition), and our macros) we processed the raw data to obtain the percentage of bacterial coverage of the optical slice at a given depth. The thickness of the pellicles and submerged biofilms was obtained by analysis of optical slices. At each time point, we determined the number of optical slices, where the percentage of bacterial coverage exceeded 10%. Both dead and viable cells were included in the analysis. The obtained monochromatic images were pseudo-coloured in Fiji ImageJ.

The morphological events during pellicle formation, growth, and development were monitored by differential interference contrast light microscopy (DIC) microscopy that enabled the visualization of pellicles in the wide-field mode. The sample preparation process was the same as for CLSM. DIC microscopy was also performed with an Axio Observer Z1 inverted microscope (Zeiss), using a ×20 magnification lens (NA 0.4). Three stacks were measured for each well. First, we sliced throughout the whole specimen (45 slices in steps of 60 μm), then we sliced the bottom of the specimen (50 slices in steps of 10 μm), and finally on the top of the preparation (100–150 slices in steps of 10 μm). The whole experiment lasted for 65 h.

For scanning electron microscopy (SEM), the pellicles of *B. subtilis* PS-216 wild type strain and mutant strains were grown in 12-well microtiter plates with a well diameter of 22.5 mm for 22 h at 37 °C. The spent culture medium was carefully removed and the samples were fixed in 2.0% (v/v) glutaraldehyde and 1.0% (v/v) paraformaldehyde in 0.1 M sodium phosphate buffer (pH 7.4) at 4 °C overnight. The chemically fixed samples were cut into smaller pieces with a scalpel, transferred to Petri dishes with a glass bottom with a diameter of 35 mm, and washed with 0.1 M sodium phosphate buffer (5 min). This procedure was repeated three times, followed by postfixation. 1% aqueous solution of OsO_4_ (w/v) was added to the samples and incubated for 1 h at 4 °C. Osmium tetraoxide was carefully removed and the samples were washed with MiliQ water (3 × 10 min). Postfixation was followed by dehydration of the samples in graded series of ethanol (50, 70, 90, 96%). The samples were left in a given ethanol concentration for 10 minutes, the liquid was removed and a higher ethanol concentration was added. At the maximum alcohol concentration (96%), the procedure was repeated 3 times. Drying of the samples was performed with the addition of hexamethyldisilazane reagent (HMDS). First, the samples were exposed for 10 min to a mixture of 96% ethanol and HMDS in a ratio of 2:1 (10 min), then in a ratio of 1:2 (10 min), and finally to the HMDS reagent alone. Samples in HMDS alone were incubated overnight in open Petri dishes at room temperature. Floating pellicle samples were attached to the metal holders with silver paint (Spi Chem, USA), coated with platinum, and observed with a JEOL JSM-7500F field-emission scanning microscope.

### Detection of spores

The presence of spores in the samples at *T*_4_ and *T*_5_ was determined using Schaeffer–Fulton’s method. In Schaeffer–Fulton’s method, a primary stain-malachite green is forced into the spore by steaming the bacterial emulsion (Sigma Aldrich protocol). Malachite green is water soluble and has a low affinity for cellular material, so vegetative cells may be decolorized with water. Vegetative cells are then counterstained with safranin.

In addition, we have examined the samples with CLSM and DIC microscopy. At each time point, 2 μl of the sample was examined under an Axio Observer Z1 (ZEISS) inverted microscope.

The fraction of spore cells in the system was determined from the ratio of spores to the total cell count. To determine the number of spores samples have been treated at 80 °C for 30 min and inoculated on agar plates. To determine the total number of vegetative cells and spores, the thermally untreated samples were inoculated on agar plates.

### Data presentation and statistical analysis

All the experiments were done in 3–6 independent biological replicates. CLSM micrographs and data sets of the most representative time series are shown. The statistical analysis and data presentation were performed in OriginPro (OriginLab, USA) program. Mean values and standard deviation were calculated. To compare mutant dynamics with the wild type, a two-sample two-sided *t* test was used. First, we calculated the variance between the samples and then applied Welch Correction to obtain the results. Compared samples showing *p* value < 0.05 were considered statistically significantly different.

### Reporting summary

Further information on research design is available in the Nature Research Reporting Summary linked to this article.

## Supplementary information

Supplementary material

Reporting Summary Checklist

## Data Availability

The authors declare that the data supporting the findings of this study are available within the paper and its Supplementary Material. Raw microscopic image data sets are available at BioStudies (EMBL-EBI) database at https://www.ebi.ac.uk/biostudies/studies/S-BIAD326 under accession number S-BIAD326. Additional data are available from the corresponding authors upon a reasonable request.

## Code availability

Macros for the determination of bacterial coverage in CLSM are available from the corresponding authors upon a reasonable request.

## References

[1] Alm E, Huang K, Arkin A (2006). The evolution of two-component systems in bacteria reveals different strategies for niche adaptation. PLoS Comput. Biol..

[2] Johnson LR (2008). Microcolony and biofilm formation as a survival strategy for bacteria. J. Theor. Biol..

[3] Pisithkul T (2019). Metabolic remodeling during biofilm development of *Bacillus subtilis*. mBio.

[4] Sretenovic S (2017). An early mechanical coupling of planktonic bacteria in dilute suspensions. Nat. Commun..

[5] da Silva SB, Cantarelli VV, Ayub MAZ (2013). Production and optimization of poly-γ-glutamic acid by *Bacillus subtilis* BL53 isolated from the Amazonian environment. Bioprocess Biosyst. Eng..

[6] Hitchman, M. L. & Gnaiger, E. In *Polarographic Oxygen Sensors* (eds. Gnaiger, E. & Hellmuth, F.) 31–36 (Springer Berlin Heidelberg, 1983).

[7] Arjes HA (2020). Biosurfactant-mediated membrane depolarization maintains viability during oxygen depletion in *Bacillus subtilis*. Curr. Biol..

[8] Jánosi IM, Kessler JO, Horváth VK (1998). Onset of bioconvection in suspensions of *Bacillus subtilis*. Phys. Rev. E.

[9] Gottenbos B, Van Der Mei HC, Nieuwenhuis P, Busscher HJ (2002). In vitro and in vivo antimicrobial activity of covalently coupled quaternary ammonium silane coatings on silicone rubber. Biomaterials.

[10] Lee LM, Rosenberg G, Rubinstein SM (2019). A sequence of developmental events occurs underneath growing *Bacillus subtilis* pellicles. Front. Microbiol..

[11] Dogsa I, Oslizlo A, Stefanic P, Mandic-Mulec I (2014). Social interactions and biofilm formation in *Bacillus subtilis*. Food Technol. Biotechnol..

[12] Vlamakis H, Chai Y, Beauregard PB, Losick R, Kolter R (2013). Sticking together: building a biofilm the *Bacillus subtilis* way. Nat. Rev. Microbiol..

[13] Kobayashi K (2007). *Bacillus subtilis* pellicle formation proceeds through genetically defined morphological changes. J. Bacteriol..

[14] Branda SS, Chu F, Kearns DB, Losick R, Kolter R (2006). A major protein component of the *Bacillus subtilis* biofilm matrix. Mol. Microbiol..

[15] Kearns DB, Chu F, Branda SS, Kolter R, Losick R (2005). A master regulator for biofilm formation by *Bacillus subtilis*. Mol. Microbiol.

[16] Dragoš A, Kovács ÁT (2017). The peculiar functions of the bacterial extracellular matrix. Trends Microbiol..

[17] Davies D (2003). Understanding biofilm resistance to antibacterial agents. Nat. Rev. Drug Discov..

[18] Boudarel H, Mathias J-D, Blaysat B, Grédiac M (2018). Towards standardized mechanical characterization of microbial biofilms: analysis and critical review. npj Biofilms Microbiomes.

[19] Jefferson KK (2004). What drives bacteria to produce a biofilm?. FEMS Microbiol. Lett..

[20] Hall-Stoodley L, Costerton JW, Stoodley P (2004). Bacterial biofilms: from the natural environment to infectious diseases. Nat. Rev. Microbiol..

[21] Branda SS, González-Pastor JE, Ben-Yehuda S, Losick R, Kolter R (2001). Fruiting body formation by *Bacillus subtilis*. Proc. Natl Acad. Sci. USA.

[22] Branda SS, Vik A, Friedman L, Kolter R (2005). Biofilms: the matrix revisited. Trends Microbiol..

[23] Flemming HC, Wingender J (2010). The biofilm matrix. Nat. Rev. Microbiol..

[24] Marvasi M, Visscher PT, Casillas Martinez L (2010). Exopolymeric substances (EPS) from *Bacillus subtilis*: polymers and genes encoding their synthesis. FEMS Microbiol. Lett..

[25] Špacapan M (2020). The ComX quorum sensing peptide of *Bacillus subtilis* affects biofilm formation negatively and sporulation positively. Microorganisms.

[26] Rubinstein SM (2012). Osmotic pressure can regulate matrix gene expression in *Bacillus subtilis*. Mol. Microbiol..

[27] Charlton SGV (2019). Regulating, measuring, and modeling the viscoelasticity of bacterial biofilms. J. Bacteriol..

[28] Kesel S (2016). Direct comparison of physical properties of *Bacillus subtilis* NCIB 3610 and B-1 biofilms. Appl. Environ. Microbiol..

[29] Qin B (2021). Hierarchical transitions and fractal wrinkling drive bacterial pellicle morphogenesis. Proc. Natl Acad. Sci. USA.

[30] Fei C (2020). Nonuniform growth and surface friction determine bacterial biofilm morphology on soft substrates. Proc. Natl Acad. Sci. USA.

[31] Yan J (2019). Mechanical instability and interfacial energy drive biofilm morphogenesis. eLife.

[32] Trejo M (2013). Elasticity and wrinkled morphology of *Bacillus subtilis* pellicles. Proc. Natl Acad. Sci. USA.

[33] Douarche C, Allain JM, Raspaud E (2015). *Bacillus subtilis* bacteria generate an internal mechanical force within a biofilm. Biophys. J..

[34] Romero D, Aguilar C, Losick R, Kolter R (2010). Amyloid fibers provide structural integrity to *Bacillus subtilis* biofilms. Proc. Natl Acad. Sci. USA.

[35] Peng N (2020). The exopolysaccharide-eDNA interaction modulates 3D architecture of *Bacillus subtilis* biofilm. BMC Microbiol..

[36] Diehl A (2018). Structural changes of TasA in biofilm formation of *Bacillus subtilis*. Proc. Natl Acad. Sci. USA.

[37] Stöver AG, Driks A (1999). Secretion, localization, and antibacterial activity of TasA, a *Bacillus subtilis* spore-associated protein. J. Bacteriol..

[38] Serrano M (1999). A *Bacillus subtilis* secreted protein with a role in endospore coat assembly and function. J. Bacteriol..

[39] Chai L (2013). Isolation, characterization, and aggregation of a structured bacterial matrix precursor. J. Biol. Chem..

[40] Hollenbeck EC (2014). Molecular determinants of mechanical properties of *V. cholerae* biofilms at the air-liquid interface. Biophys. J..

[41] Rühs, P. A., Böni, L., Fuller, G. G., Inglis, R. F. & Fischer, P. In-situ quantification of the interfacial rheological response of bacterial biofilms to environmental stimuli. *PLoS ONE* **8**, e78524 (2013). 10.1371/journal.pone.0078524 PMC3823922 24244319

[42] Pandit S (2020). The exo-polysaccharide component of extracellular matrix is essential for the viscoelastic properties of *Bacillus subtilis* biofilms. Int. J. Mol. Sci..

[43] Hollenbeck EC (2016). Mechanical behavior of a *Bacillus subtilis* pellicle. J. Phys. Chem. B.

[44] Jana S (2020). Nonlinear rheological characteristics of single species bacterial biofilms. npj Biofilms Microbiomes.

[45] Yannarell SM, Grandchamp GM, Chen SY, Daniels KE, Shank EA (2019). A dual-species biofilm with emergent mechanical and protective properties. J. Bacteriol..

[46] Klapper I, Rupp CJ, Cargo R, Purvedorj B, Stoodley P (2002). Viscoelastic fluid description of bacterial biofilm material properties. Biotechnol. Bioeng..

[47] Peterson BW (2015). Viscoelasticity of biofilms and their recalcitrance to mechanical and chemical challenges. FEMS Microbiol. Rev..

[48] Pen Y (2015). Effect of extracellular polymeric substances on the mechanical properties of *Rhodococcus*. Biochim. Biophys. Acta Biomembr..

[49] Stefanic P, Mandic-Mulec I (2009). Social interactions and distribution of *Bacillus subtilis* pherotypes at microscale. J. Bacteriol..

[50] Durrett R (2013). Genome sequence of the *Bacillus subtilis* biofilm-forming transformable strain PS216. Genome Announc..

[51] Spacapan M, Danevčič T, Mandic-Mulec I (2018). ComX-induced exoproteases degrade ComX in *Bacillus subtilis* PS-216. Front. Microbiol..

[52] Oslizlo A (2015). Exploring ComQXPA quorum-sensing diversity and biocontrol potential of *Bacillus* spp. isolates from tomato rhizoplane. Microb. Biotechnol..

[53] Stefanic P, Kraigher B, Lyons NA, Kolter R, Mandic-Mulec I (2015). Kin discrimination between sympatric *Bacillus subtilis* isolates. Proc. Natl Acad. Sci. USA.

[54] Kalamara M, Spacapan M, Mandic-Mulec I, Stanley-Wall NR (2018). Social behaviours by *Bacillus subtilis*: quorum sensing, kin discrimination and beyond. Mol. Microbiol..

[55] Dogsa I (2021). Peptide signaling without feedback in signal production operates as a true quorum sensing communication system in *Bacillus subtilis*. Commun. Biol..

[56] Lyons NA, Kolter R (2018). A single mutation in *rapP* induces cheating to prevent cheating in *Bacillus subtilis* by minimizing public good production. Commun. Biol..

[57] Mutlu A, Kaspar C, Becker N, Bischofs IB (2020). A spore quality–quantity tradeoff favors diverse sporulation strategies in *Bacillus subtilis*. ISME J..

[58] Danevčič T (2021). Surfactin facilitates horizontal gene transfer in *Bacillus subtilis*. Front. Microbiol..

[59] Stefanic P (2021). Kin discrimination promotes horizontal gene transfer between unrelated strains in *Bacillus subtilis*. Nat. Commun..

[60] Miras M, Dubnau D (2016). A DegU-P and DegQ-dependent regulatory pathway for the K-state in *Bacillus subtilis*. Front. Microbiol..

[61] Bridier, A. et al. The spatial architecture of *Bacillus subtilis* biofilms deciphered using a surface-associated model and in situ imaging. *PLoS ONE* **6**, e16177 (2011). 10.1371/journal.pone.0016177 PMC3022735 21267464

[62] Chai Y, Kolter R, Losick R (2010). Reversal of an epigenetic switch governing cell chaining in *Bacillus subtilis* by protein instability. Mol. Microbiol..

[63] Dogsa I, Brloznik M, Stopar D, Mandic-Mulec I (2013). Exopolymer diversity and the role of levan in *Bacillus subtilis* biofilms. PLoS ONE.

[64] Sella SRBR, Vandenberghe LPS, Soccol CR (2014). Life cycle and spore resistance of spore-forming *Bacillus atrophaeus*. Microbiol. Res..

[65] McLoon AL, Guttenplan SB, Kearns DB, Kolter R, Losick R (2011). Tracing the domestication of a biofilm-forming bacterium. J. Bacteriol..

[66] Powers MJ, Sanabria-Valentín E, Bowers AA, Shank EA (2015). Inhibition of cell differentiation in *Bacillus subtilis* by *Pseudomonas protegens*. J. Bacteriol..

[67] Lyons NA, Kraigher B, Stefanic P, Mandic-Mulec I, Kolter R (2016). A combinatorial kin discrimination system in *Bacillus subtilis*. Curr. Biol..

[68] Soo-Gun OH, Slattery JC (1978). Disk and biconical interfacial viscometers. J. Colloid Interface Sci..

[69] Erni P (2003). Stress- and strain-controlled measurements of interfacial shear viscosity and viscoelasticity at liquid/liquid and gas/liquid interfaces. Rev. Sci. Instrum..

[70] Tajuelo J, Rubio MA, Pastor JM (2018). Flow field based data processing for the oscillating conical bob interfacial shear rheometer. J. Rheol..

[71] Boussinesq JM (1913). The application of the formula for surface viscosity to the surface of a slowly falling droplet in the midst of a large unlimited amount of fluid which is at rest and possesses a smaller specific gravity. Ann. Chem. Phys..

[72] Bridier A, Meylheuc T, Briandet R (2013). Realistic representation of *Bacillus subtilis* biofilms architecture using combined microscopy (CLSM, ESEM and FESEM). Micron.

[73] Oknin H, Steinberg D, Shemesh M (2015). Magnesium ions mitigate biofilm formation of *Bacillus* species via downregulation of matrix genes expression. Front. Microbiol..

[74] Cao H (2016). Revealing region-specific biofilm viscoelastic properties by means of a micro-rheological approach. npj Biofilms Microbiomes.

[75] Dergham Y (2021). Comparison of the genetic features involved in *Bacillus subtilis* biofilm formation using multi-culturing approaches. Microorganisms.

