# Supplementary material for: Systems view of Bacillus subtilis pellicle development
Source: NPJ Biofilms Microbiomes. 2022 Apr 12;8:25. doi: 10.1038/s41522-022-00293-0 (PMC9005697; doi:10.1038/s41522-022-00293-0)
Supplement: Supplementary file 1 — Supplementary material [file 41522_2022_293_MOESM1_ESM.pdf]

SUPPLEMENTARY INFORMATION

Supplementary Table 1: *Bacillus subtilis* strains used in this study.

***B. subtilis* strains**	**Genetic background**	**Reference**
B. subtilis PS-216 wt (unlabelled)	undomesticated wild type isolate	[1]
BM1315 *B. subtilis* PS-216 wt	*amyE::*p$_{hyper}$*cl*$_0$*3-yfp* (Sp)	this study
DL963 *B. subtilis* NCIB 3610 (Δ*tasA*)	*B. subtilis* NCIB 3610<br>Δ*tasA:: spec* (Sp)	[2]
BM1882 *B. subtilis* PS-216 (Δ*tasA*)	Δ*tasA:: spec* (Sp)<br>*sacA::* p43-*yfp* (Cm)	this study
ZK4300 *B. subtilis* NCIB 3610 (Δ*epsA-O*)	*B. subtilis* NCIB 3610<br>Δ*epsA-O::tet* (Tc)	[3]
BM1311 *B. subtilis* PS-216 (Δ*epsA-O*)	Δ*epsA-O::tet* (Tc)<br>*amyE::*p$_{hyper}$*cl*$_0$*3-yfp* (Sp)	this study
BM1844 *B. subtilis* PS-216 (Δ*tasA* Δ*epsA-O* unlabelled)	Δ*tasA::spec* (Sp)<br>Δ*epsA-O::tet* (Tc)	Podnar et al., unpublished
BM1926 *B. subtilis* PS-216 (Δ*tasA* Δ*epsA-O*)	Δ*tasA::spec* (Sp)<br>Δ*epsA-O::tet* (Tc)<br>*sacA::* p43-*yfp* (Cm)	this study
E. coli plasmid	**Genetic background**	**Reference**
pEM1071	DH5α *sacA*::p43-*yfp* (Cm), Amp	[4]

Supplementary Note 1: Optimization of vertical profile scanning.

Commercial microscopes enable the user an automatic adjustment of many parameters, but they cannot take into account the quality of the acquired image, the information about distribution, nor the time taken to carry out the complex microscope experiment like in our case. To describe the crucial events during biofilm formation we wanted to achieve a time resolution of < 1h, therefore the default settings had to be optimized. This meant scarifying less important information details for exchange in speed. We have therefore optimized the pinhole size. The used setting of the pinhole (97 μm) improved the acquired emitted light intensity significantly at an optical slice thickness of 11 μm. Image resolution was 512 x 512. Pixel size corresponded to 1.6 μm, enough to capture a single *B. subtilis* cell.

Optimizing sample slicing: The first stack was a coarse sliced stack: with a step of 60 μm through the entire height of the specimen (about 2700 μm) = 50 slices; the second stack was fine sliced focused at the bottom of the specimen (up to 500 μm height): with a step of 10 μm = 50 slices; the third stack was the upper part of the sample (at the water-air interface, 1000-1500 μm), which was fine sliced with a step of 10 μm = 100-150 slices. The coarse slicing was used to obtain an overview of the system. As no special structures appeared to be formed in the middle of the sample and the images in the middle of the sample did not change significantly from one to another slice 60 μm apart, we decided to use these acquired images to represent the middle part of the sample.

The total number of slices acquired per well was thus 215. After optimization, it took 273 s to scan a single well and in average 60 min to scan all 12 wells.

Laser settings: In the microscopy analysis results were presented as % coverage by YFP-cells or propidium iodide-stained cells per slice. We assumed that the number of the cells was proportional to their total area. The fluorescence intensity of the objects, which is a measure

for the concentration of fluorochromes was not considered here. What we were considering was the ratio of areas, rather than the ratio of intensities. The intensity of the emitted fluorescence has to be above the threshold, to distinguish cells from the background and low enough to prevent overexposure during all incubation time points in all slices. This is fluorochrome (& optics) dependent. Therefore, the laser settings in the first slice were different for the two fluorochromes (green 1.5 %; red 1.0 %).

As the excitation and emission light travel through the medium some losses (i.e. absorption, scattering) occur, therefore, to acquire the same amount of emission light from the objects deeper in the sample one requires more excitation light. We used a linear extrapolation model, which we calibrated to the autofluorescence of the inoculated media at time zero. After setting the laser intensity at the top of the sample, we sliced through the sample, to check that the image intensity was constant in all slices, regardless of the slice position in z-axis. During the calibration procedure, we ensured that the objects lying at the bottom of the specimen appeared equally intense as those on the top of the sample. The laser intensity on the top of the sample was (3.5 % for green and 2.6 % for red) and the scaling-up factor of the two lasers was not exactly the same (2.3 x for green, 2.6 x for red). This relatively small difference suggests that the loss of excitation/emission light was slightly different for the two fluorescent channels used, which we took into account by our calibration procedure.

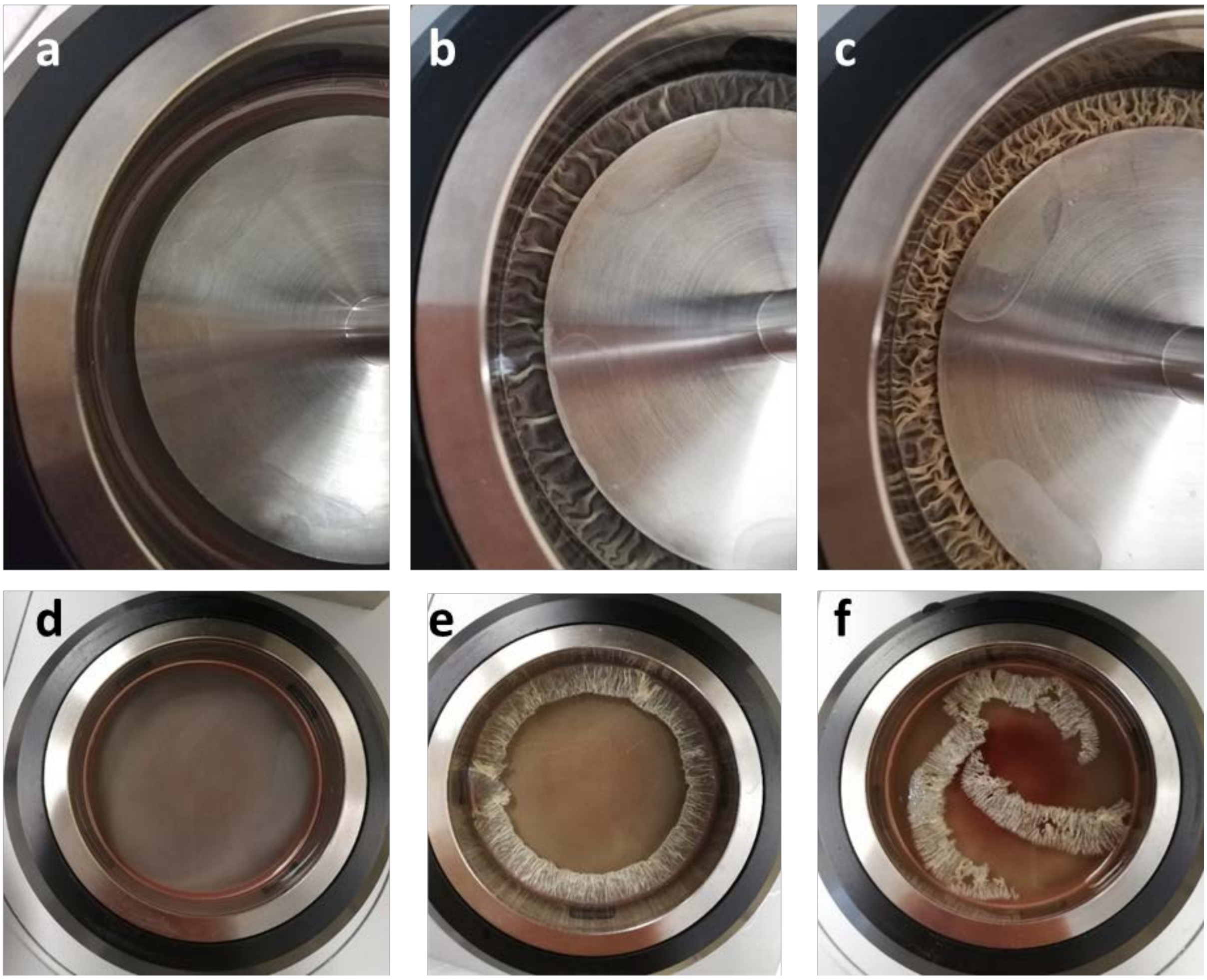

Supplementary Figure 1: Pellicles of *Bacillus subtilis* PS 216 wild type strain in the IRS dish. (**a)** IRS dish at the beginning of incubation, (**b**) after 24 h, and (**c)** after 65 h of incubation. Figures **d-f** show pellicles after the measuring system was lifted. Due to wrinkle formation, the contact of the pellicle with the measuring system could be reduced which in turn would decrease the measured G′ values. The pellicle disintegrated after removal of the measuring system (Supplementary Figure 1f); the end of the experiment correlated with measured decrease in G′.

Supplementary Table 2: Key events during pellicle development for different bacterial strains. T_0-T_5 denotes key event points when marked changes in viscoelastic behaviour at the water-air interface occurred. Results were obtained from the raw data obtained by the interfacial rheology method and are presented as means ± standard deviation from 3-6 independent biological measurements. Stars denote statistically significant differences in mutant pellicles compared to PS-216 wt ($p < 0.05$).

**Bacterial strain**	**T_0 [h]**	**T_1 [h]**	**T_2 [h]**	**T_3 [h]**	**T_4 [h]**	**T_5 [h]**
PS-216 wt	8.9 ± 0.3	11.4 ± 0.2	12.6 ± 0.1	23.4 ± 1.5	29.4 ± 1.5	50.0 ± 0.5
PS-216 Δ*tasA*	9.3 ± 0.1 *	11.0 ± 0.3	14.0 ± 0.3 *	23.1 ± 0.2	27.2 ± 0.4 *	55.4 ± 0.1 *
PS-216 Δ*epsA-O*	8.3 ± 0.2 *	10.0 ± 0.3 *	15.6 ± 1.5 *	25.3 ± 1.1	30.5 ± 0.5	57.1 ± 1.3 *
PS-216 Δ*tasA* Δ*epsA-O*	9.0 ± 0.2	11.5 ± 0.2	14.7 ± 0.5 *	22.8 ± 0.6	34.7 ± 1.5 *	59.6 ± 1.0 *

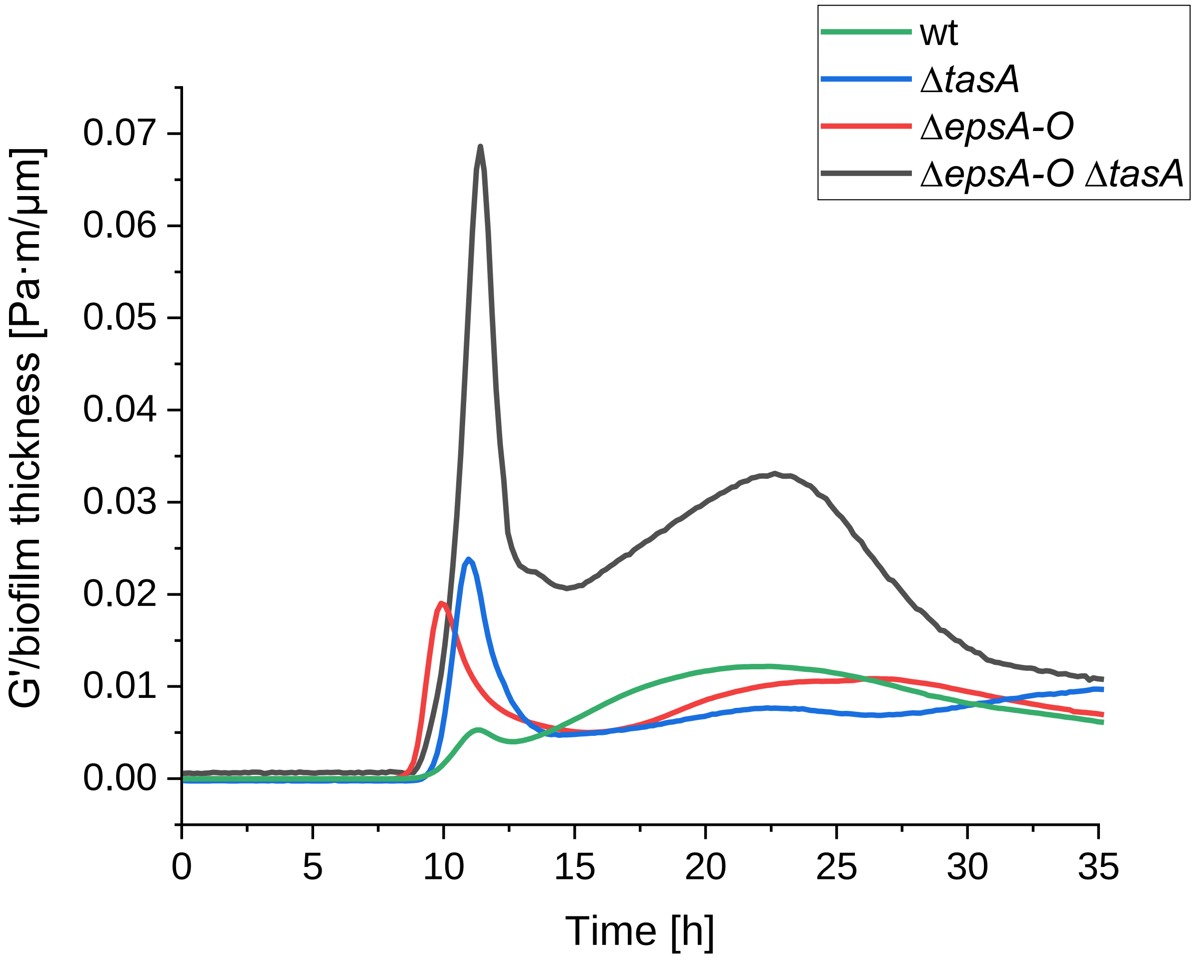

Supplementary Figure 2: Interfacial storage modulus normalised to the maximum biofilm thickness. Viscoelastic curves are presented for *B. subtilis* PS-216 wild type strain (green), Δ*tasA* (blue), Δ*epsA-O* (red), and Δ*epsA-O* Δ*tasA* (black) mutant strains as a function of time during the pellicle developmental process at the water-air interface in MSgg medium. This normalization was made with an assumption of a homogeneous elastic profile across the pellicle thickness. Results are presented as mean from 3-6 independent biological measurements.

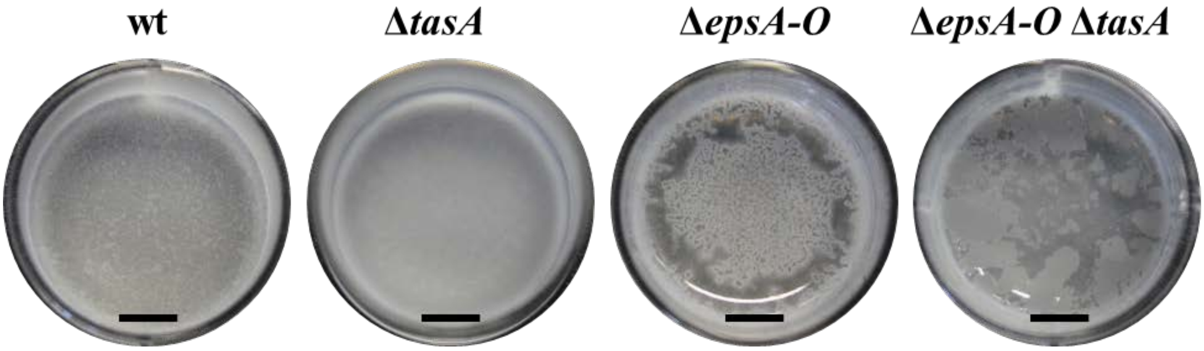

Supplementary Figure 3: Images of the pellicles grown in 12-well microtiter plates. Pellicles in MSgg liquid medium after 22 h of incubation at 37 °C for *B. subtilis* PS-216 wild type strain, Δ*tasA*, Δ*epsA-O,* and Δ*epsA-O* Δ*tasA* mutant strains are shown. Images were taken with a digital camera Canon EOS 600D. Scale bar represents 5 mm.

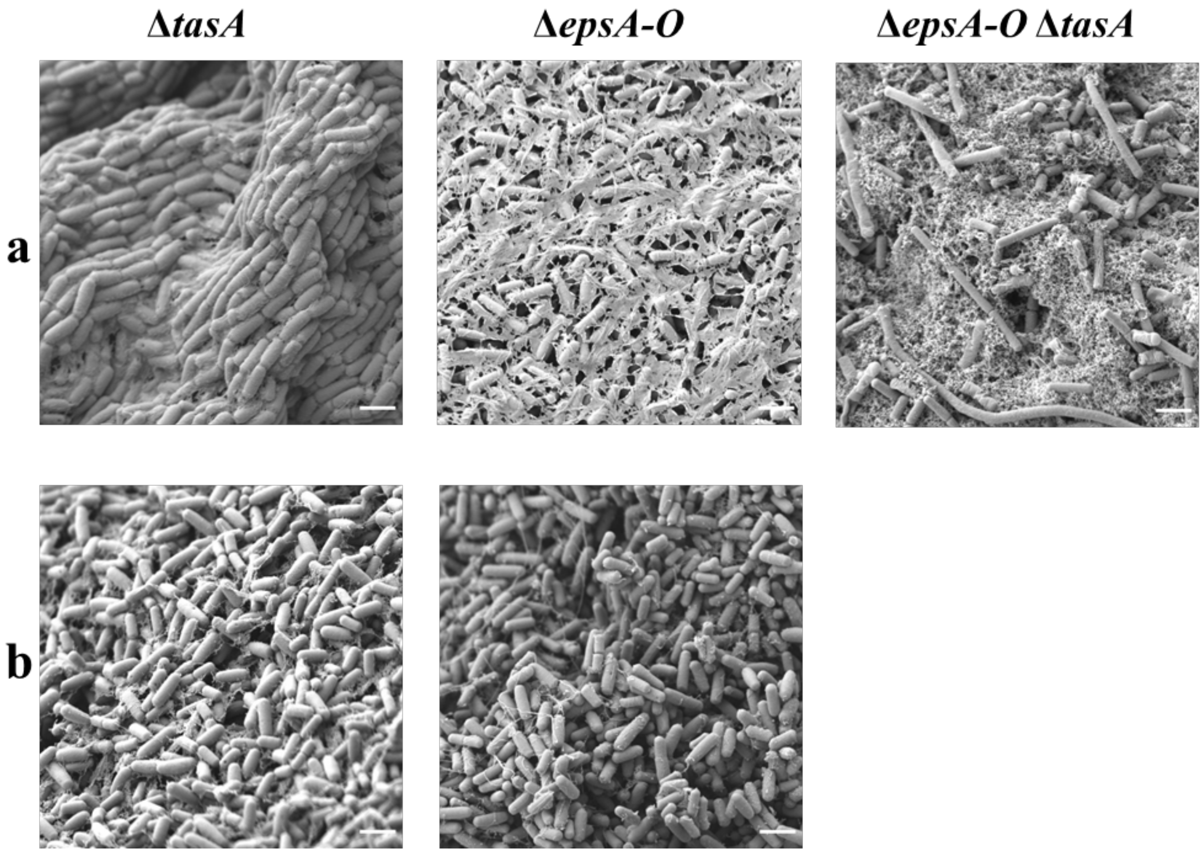

Supplementary Figure 4: Scanning electron micrographs of *B. subtilis* PS-216 mutant strain pellicles. (**a**) Micrographs of the top layer in contact with air. (**b**) Micrographs of the bottom layer of the pellicle in contact with the liquid MSgg medium. Due to the thin nature of the double mutant pellicle, only the top surface is presented. Scale bars represent 2 µm.

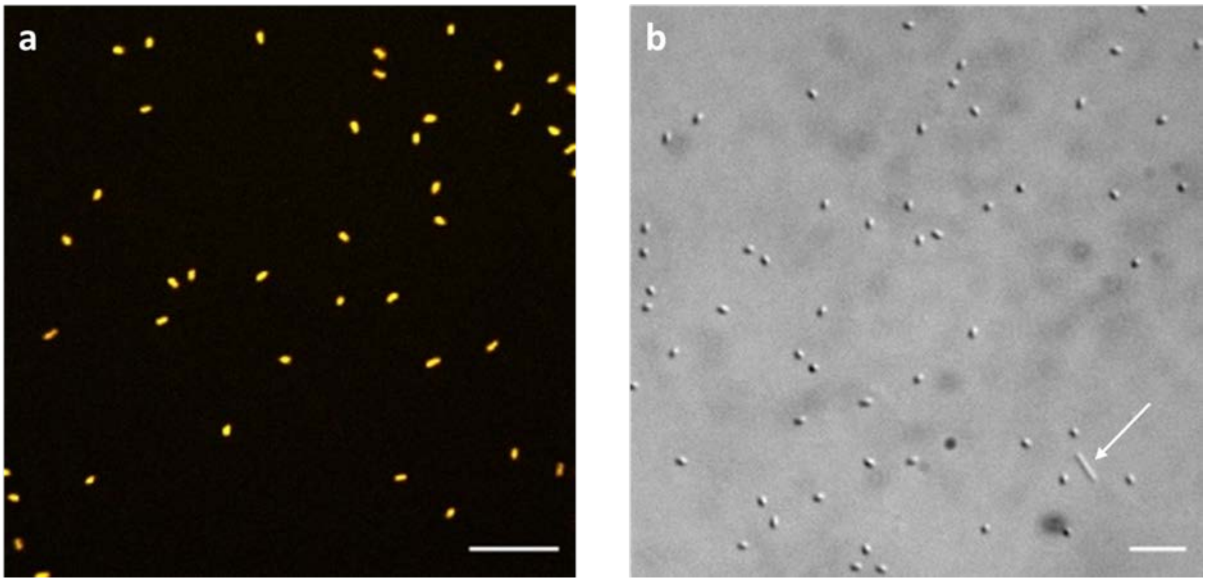

Supplementary Figure 5: Detection of spores at T_5. (**a**) CLSM micrograph of YFP fluorescence emitting spores. (**b)** DIC micrograph of bacterial spores and bacterial cell (white arrow) at T_5. Scale bars represent 10 µm.

a

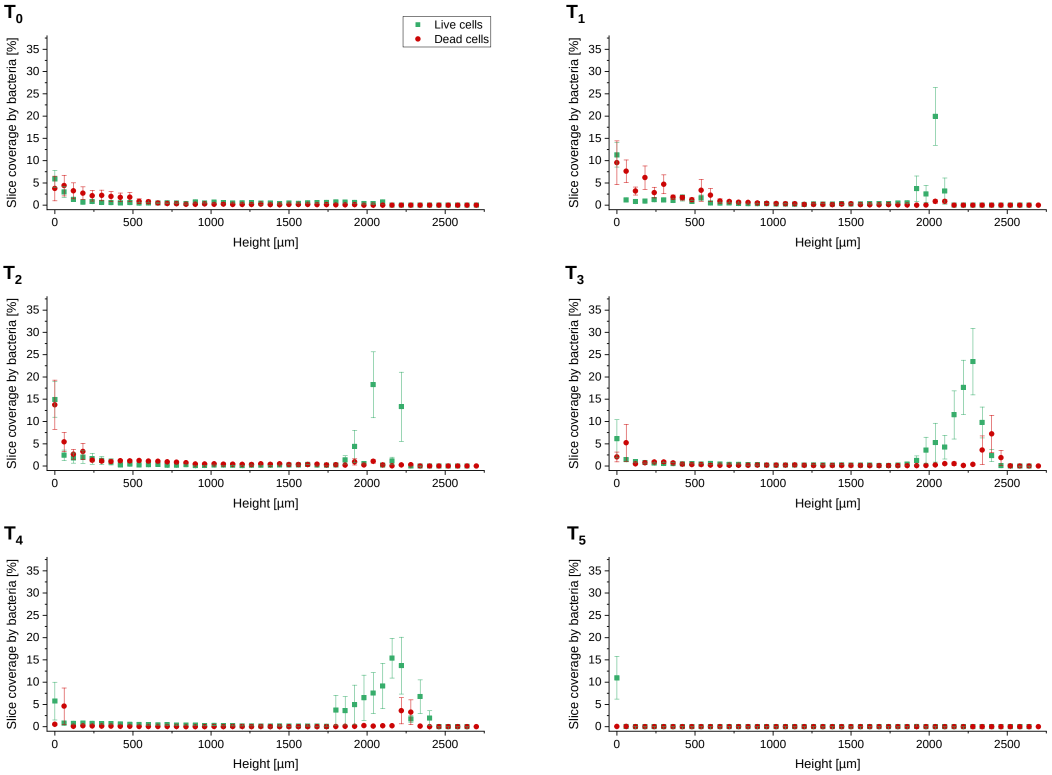

T_0
T_1
T_2
T_3
T_4
T_5
Live cells
Dead cells
Slice coverage by bacteria [%]
Height [µm]
0
5
10
15
20
25
30
35
0
500
1000
1500
2000
2500

b

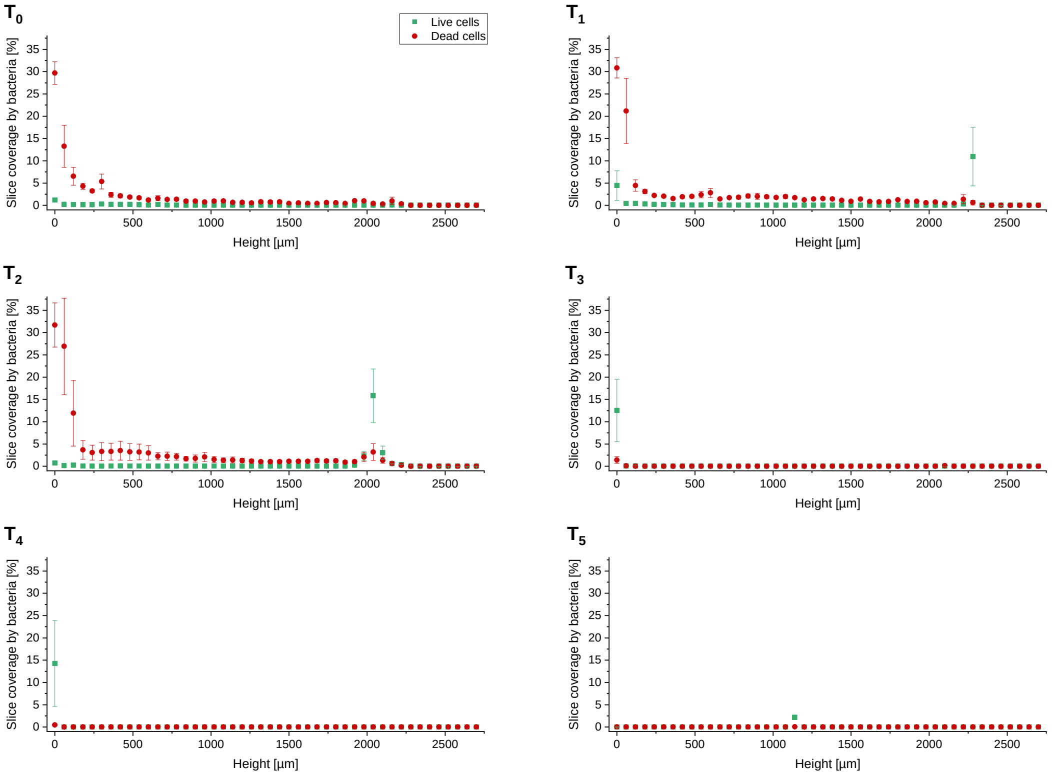

T_0
T_1
T_2
T_3
T_4
T_5
Live cells
Dead cells
Slice coverage by bacteria [%]
Height [µm]
0
5
10
15
20
25
30
35
0
500
1000
1500
2000
2500

c

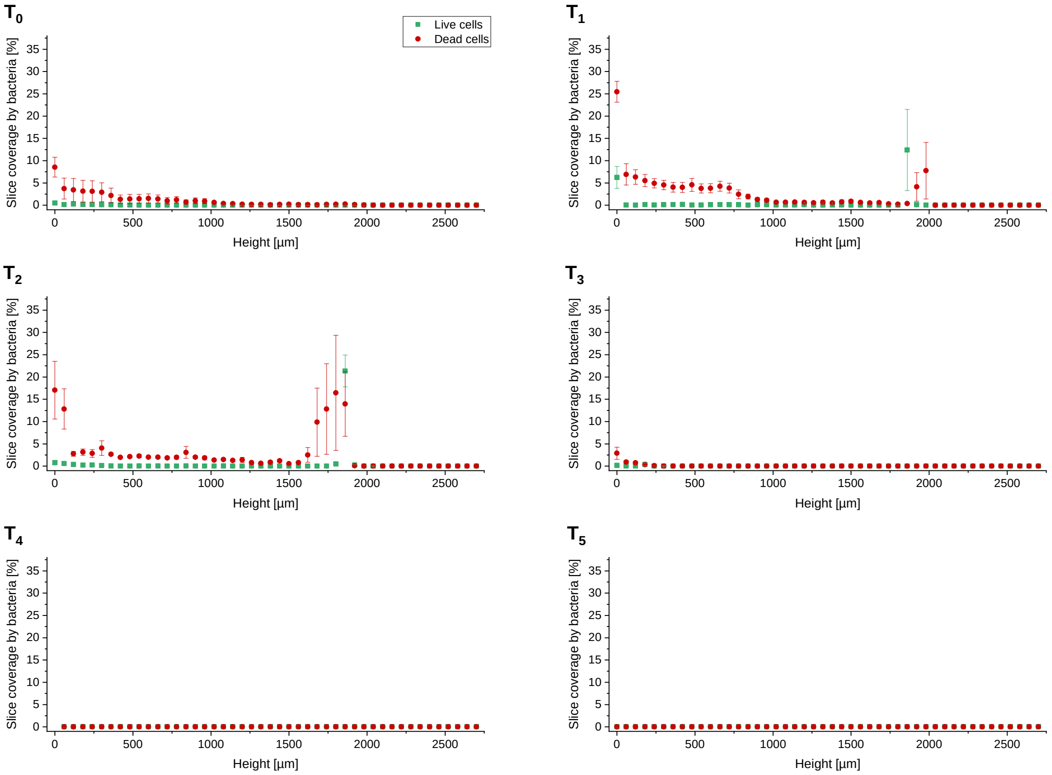

T$_0$
T$_1$
T$_2$
T$_3$
T$_4$
T$_5$
Live cells
Dead cells
Slice coverage by bacteria [%]
Height [µm]
0
5
10
15
20
25
30
35
500
1000
1500
2000
2500

d

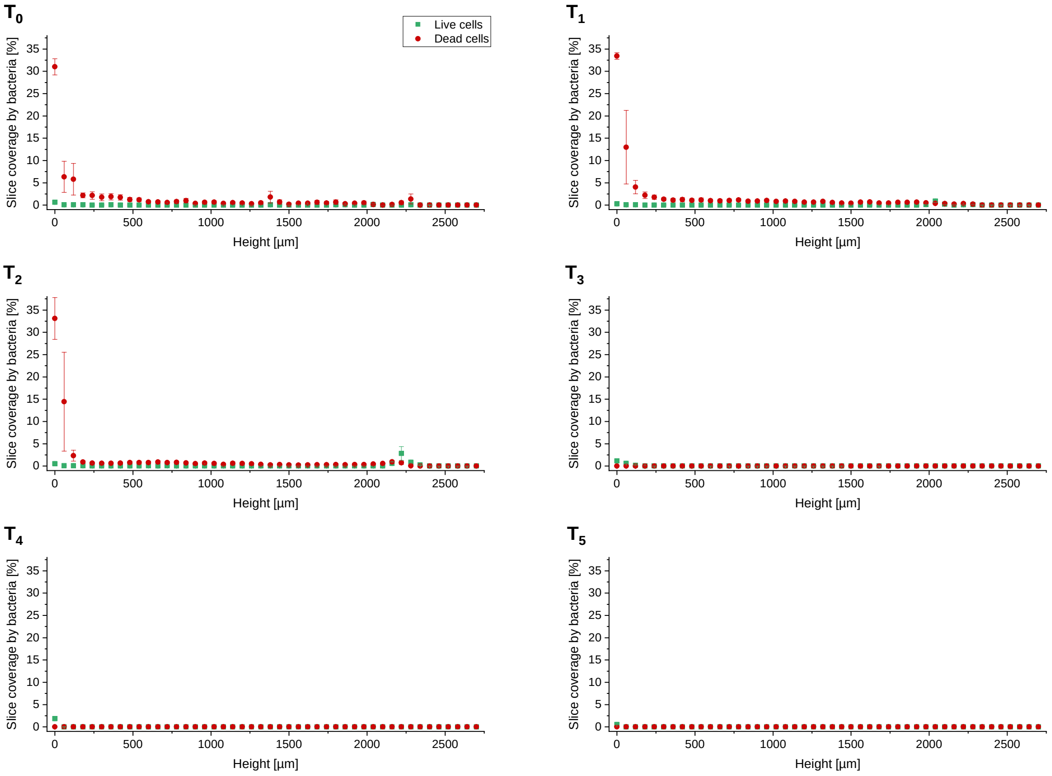

T$_0$
T$_1$
T$_2$
T$_3$
T$_4$
T$_5$
Live cells
Dead cells
Slice coverage by bacteria [%]
Height [µm]
0
5
10
15
20
25
30
35
500
1000
1500
2000
2500

Supplementary Figure 6: Temporal distribution of viable and dead cells along the vertical profile. Temporal distribution is presented for T_0-T_5 time points in *B. subtilis* PS-216 wild type strain (**a**), Δ*tasA* (**b**), Δ*epsA-O* (**c**), and Δ*epsA-O* Δ*tasA* (**d**) mutants. Viable cells were constitutively expressing YFP, while the red cells with compromised membranes were stained with PI. Results are presented as means ± standard error from 3-6 independent biological measurements.

Supplementary References

1. Stefanic, P. & Mandic-Mulec, I. Social interactions and distribution of *Bacillus subtilis* pherotypes at microscale. *J. Bacteriol.* **191**, 1756–1764 (2009).

2. Branda, S. S., Chu, F., Kearns, D. B., Losick, R. & Kolter, R. A major protein component of the *Bacillus subtilis* biofilm matrix. *Mol. Microbiol.* **59**, 1229–1238 (2006).

3. Lyons, N. A., Kraigher, B., Stefanic, P., Mandic-Mulec, I. & Kolter, R. A combinatorial kin discrimination system in *Bacillus subtilis*. *Curr. Biol.* **26**, 733–742 (2016).

4. Stefanic, P. *et al.* Kin discrimination promotes horizontal gene transfer between unrelated strains in *Bacillus subtilis*. *Nat. Commun.* **12**, 3457 (2021).
